# Pond Water eDNA Reflects Broad Consistency with Surrounding Terrestrial Plant Ecosystems

**DOI:** 10.3390/biology14010062

**Published:** 2025-01-13

**Authors:** Duygu Bozdogan, Shogo Takizawa, Norihiro Furukori, Kosuke Homma, Harue Abe, Hitoshi Sakio, Naoki Harada, Kazuki Suzuki

**Affiliations:** 1 Graduate School of Science and Technology, Niigata University, Niigata 950-2181, Japan; 2 Faculty of Agriculture, Niigata University, Niigata 950-2181, Japan; 3 Sado Island Center for Ecological Sustainability, Niigata University, Niigata 952-0103, Japan; 4 Institute of Science and Technology, Niigata University, Niigata 950-2181, Japan

**Keywords:** ecological monitoring, eDNA, plant community, fresh water, filtration

## Abstract

This study evaluates the potential of using pond water eDNA to reflect the surrounding terrestrial plant communities, aiming to develop a sustainable, large-scale, and long-term monitoring method for plant diversity in forest ecosystems. Water samples were collected four times from two ponds with different vegetation types during the late spring to autumn seasons in Japan. eDNA was extracted from dissolved particles fractionated by sequential filtration through pore sizes of 200 µm, 5 µm, and 0.45 µm, followed by high-throughput amplicon sequencing targeting the plant rbcL gene. By comparing field surveys with the eDNA data, we identified 79% and 63% of plant families and genera, respectively, suggesting that pond water eDNA may reflect the surrounding terrestrial plant ecosystem. Additionally, different trends were observed in the seasonal variation of plant taxa and their composition detected in eDNA, based on particle size. This study highlights the potential of pond water eDNA to provide valuable insights into forest plant richness and seasonal dynamics, offering a novel approach for ecological monitoring.

## Simple Summary

The species composition of terrestrial plant ecosystems has traditionally been assessed through field surveys, but the need for specialized knowledge in morphological identification and the considerable effort involved raise concerns about the sustainability of large-scale and long-term monitoring. In this study, we explored the potential of using pond water eDNA to serve as an indicator of the surrounding terrestrial plant ecosystem’s species composition. The results indicated that the plant composition detected from the pond water eDNA closely reflected that of the surrounding terrestrial plant ecosystem, suggesting this method’s potential application as an alternative monitoring tool.

## 1. Introduction

Detecting the composition changes in a plant community is crucial for understanding the ecosystem health and developing sustainable management strategies. Previous studies have made it clear that plant species diversity and total biomass influence ecosystem function and stability [1,2]. The forest ecosystem is a typical reservoir of biodiversity and essential provider of ecosystem services, including carbon absorption, water regulation, and soil stabilization [3]. Therefore, monitoring species diversity and the biomass of terrestrial plant communities in relation to environmental factors is important for identifying vulnerabilities and designing adaptive forest management strategies [4]. Modern research techniques such as remote sensing and geographic information systems (GIS) [5] have increasingly been utilized in forest ecosystem monitoring [6], especially for estimating carbon stock. These techniques still face challenges related to data quality, atmospheric conditions, topographic complexities, and sensor calibration, with the reliability of the model depending on the data quality, which in turn influences the accuracy of the results [7,8,9]. However, they offer the advantage of enabling large-scale and long-term monitoring of forest ecosystem biomass at a lower cost and with reduced labor requirements. On the other hand, while these techniques are fundamentally useful for obtaining data indicative of biomass, they are inherently incapable of serving as indicators of species composition. The traditional approach to investigating species composition in forest ecosystems involves field surveys with species identification using specimens. However, long-term, large-scale monitoring requires substantial costs, effort, and expertise in morphological identification, presenting challenges to its sustainability. Consequently, the application of environmental DNA (eDNA) metabarcoding has gained increasing attention as a promising method for sustainable biodiversity monitoring in recent years across various ecosystems [10,11].

eDNA analysis utilizes genes left by organisms in the environment to detect the presence of species. The approaches offer comprehensive results for the genetic identification of a large number of organisms with minimal effort, without requiring advanced expertise, and is particularly beneficial for detecting plants with small biomass that are difficult to observe directly [12]. Today, eDNA analysis is effectively applied in diverse objectives, such as marine and freshwater ecology [13], paleoecology [14], structural food webs [15], and various terrestrial biospheres, including extreme environments [16]. It serves important roles in investigating biodiversity, monitoring invasive species, and evaluating ecological restoration efforts [17]. In aquatic environments, eDNA analysis has been reported to be useful for detecting fish, amphibians, and invertebrates; however, the applications of detecting and monitoring terrestrial plant species are limited. In Banerjee et al. [18], 4114 eDNA studies were reviewed and it was found that only 558 (13%) of them focused on plant species or communities. Furthermore, only 4% of them focused on vascular plants, with many applications related to aquatic plants and pollen analysis of paleoecology. To our knowledge, there are very few instances of eDNA being used to monitor current terrestrial plant ecosystems.

The forest plant ecosystem is increasingly being studied and managed at the watershed scale. Ponds are small aquatic ecosystems where materials transported by water flow from the surrounding watershed accumulate, creating distinct environments. This characteristic supports higher biodiversity compared to larger freshwater habitats such as torrents and rivers [19]. Serving as refuges for many species, ponds host numerous aquatic plant species [20]. In particular, ponds serve as ecological connectors between aquatic and terrestrial ecosystems, making them invaluable for studying biodiversity patterns across these environments [21]. Additionally, ponds work as natural reservoirs for genetic materials from the surrounding terrestrial environments, accumulated through water flow and precipitation, thereby capturing a broad spectrum of eDNA [22]. It was reported that eDNA signals from terrestrial mammals surrounding the pond, although weaker than those from semi-aquatic mammals, can also be detected from the pond water [23]. Furthermore, it was reported that terrestrial plants, as well as aquatic plants, were detected in water eDNA from small lakes, and that lake eDNA is relatively homogeneous between offshore and nearshore samples [24]. In addition, it has been reported that ponds provide a protective environment for DNA from ultraviolet radiation and drastic temperature fluctuations [25]. Considering these findings, ponds in forests could effectively indicate terrestrial plant biodiversity and are therefore considered the best sampling sites for examining the effectiveness of eDNA analysis in understanding the surrounding plant vegetation.

The purpose of this study is to evaluate whether pond water eDNA reflects the surrounding terrestrial plant communities. If this approach proves effective, it could contribute to the development of a sustainable long-term and large-scale monitoring method for plant species diversity in forest ecosystems. Based on the reasons mentioned above, we hypothesized that pond water eDNA reflects the surrounding terrestrial plant ecosystems. We will test this hypothesis using pond water eDNA analysis and vegetation surveys.

## 2. Materials and Methods

### 2.1. Study Sites and Sample Collection

**Figure 1:**
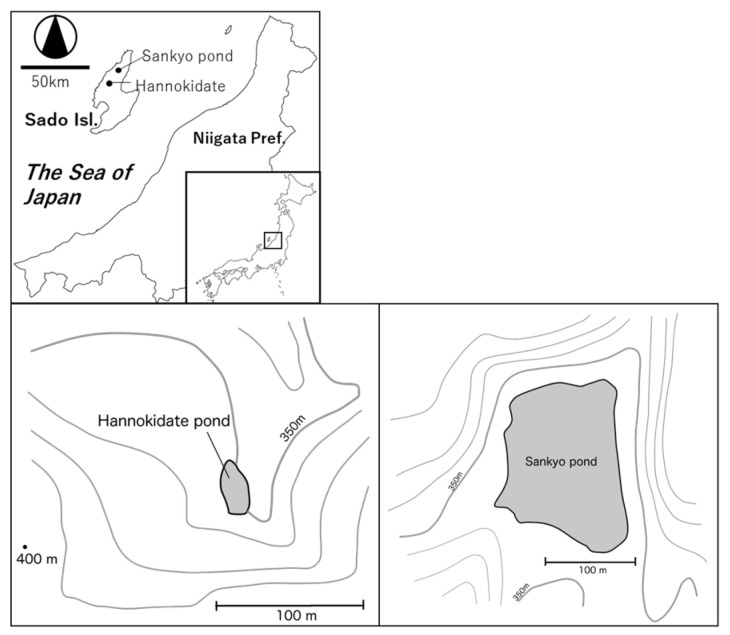
Location of study area and sampling sites. Map showing the location of two ponds where samples were collected. The map includes contour lines indicating elevation.

In this study, we selected two ponds, Sankyo Pond (38.27849° N, 138.47022° E) and Hannokidate Pond (38.20201° N, 138.43344° E), located in Sado City, Niigata Prefecture, Japan (Figure 1), a region with well-documented plant biodiversity. Both ponds are located in the mid-slope of Osado Mountain (with an average elevation of approximately 900 m) at the same elevation band (around 350 m), and are relatively shallow, with depths of less than 3 m. They are characterized by the absence of visible inflows and outflows, with no discernible river or stream feeding into them from upstream. The surrounding vegetation of Sankyo Pond consists of a secondary deciduous broad leaf forest, dominated by *Quercus serrata*, whereas that of Hannokidate Pond is an evergreen coniferous forest, dominated by *Thujopsis dolabrata* var. *hondae*, *Alnus japonica*, and planted Japanese cedar (*Cryptomeria japonica*). A vegetation survey by visual inspection was conducted on 21 September 2023.

In 2023, water samples were collected from the ponds on 15 May, 26 July, 21 September, and 13 November, on days with no rain, covering spring to late autumn. Surface water samples (2 L each) were collected from three fixed points within each pond during each sampling event using a ladle. These three samples from each pond, collected on the same day, were treated as replicates. The samples were stored in sterile plastic bags, and two mL of benzalkonium chloride solution, known for its effectiveness in preserving DNA, was added to each sample, resulting in a final concentration of 0.01%. The water samples were then stored at 4 °C until further filtration processes.

### 2.2. DNA Extraction from the Water Samples

The plant-derived materials containing DNA in the pond water are anticipated to include relatively large plant fragments, pollen, finely degraded tissues, and chloroplasts. These materials vary in size by approximately one order of magnitude. Therefore, the water sample was processed using a three-stage sequential filtration to recover plant DNA based on the particle size distribution in the pond water. In the first stage, a 200 µm nylon mesh (Tantore Co., Ltd., Aichi, Japan) was used to separate the coarse organic fraction. Next, the filtrate was passed through a 5 µm membrane filter (Toyo Roshi Kaisha, Ltd., Tokyo, Japan) to capture fine suspended matter such as pollen. Subsequently, the filtrate was further passed through a 0.45 µm Sterivex filter (Merck KGaA, Darmstadt, Germany) to recover the remaining eDNA. After passing through the Sterivex filter, 1.6 mL of RNAlater (Thermo Fisher Scientific, Waltham, MA) was added to the Sterivex filter. These samples were stored in a −20 °C freezer until DNA extraction.

DNA extraction from the mesh and membrane filters was performed using ISOIL for Beads Beating (Nippon Gene, Tokyo, Japan). The extraction procedure followed the manufacturer’s instructions, utilizing FastPrep 24 (MP-Biomedicals, Irvine, CA, USA) as the bead cell lysis device with shaking conditions set at 4.0 m second^−1^ for 45 s. DNA extraction from the Sterivex filters was performed using the DNeasy Blood & Tissue Kit (Qiagen, Hilden, Germany), following the manufacturer’s instructions. Subsequently, the extracted DNA was stored at −20 °C.

### 2.3. Sequencing Process

The primer pairs rbcL_F3/rbcL_R4 [26] were applied to amplify the plant rbcL (ribulose 1,5-bisphosphate carboxylase/oxygenase L subunit) gene sequences. Using TaKaRa EX premier DNA polymerase (Takara-Bio, Kusatsu, Japan), PCR amplification was performed under the following conditions: initial denaturation at 94 °C for 1 min, followed by 40 cycles of denaturation at 98 °C for 10 s, annealing at 55 °C for 15 s, and extension at 68 °C for 30 s, with a final extension at 68 °C for 3 min. Subsequently, the PCR products were purified using Agencourt AMPure XP (Beckman Coulter, Brea, CA, USA) according to the manufacturer’s instructions. Index PCR was then performed to attach indices to both ends of the first PCR products using primers from the Nextera XT Index kit (Illumina, San Diego, CA, USA). The PCR conditions for the index PCR were as follows: initial denaturation at 94 °C for 3 min, followed by denaturation at 98 °C for 30 s, annealing at 55 °C for 30 s, and extension at 68 °C for 30 s, with an additional 12 cycles of annealing and extension at 68 °C for 30 s each, and a final extension at 68 °C for 5 min. The purified amplicons were paired-end sequenced on an Illumina MiSeq Platform at a read length of 2 × 300 bp using the MiSeq reagent kit v3 (Illumina). The sequence data have been deposited in the DNA Data Bank of Japan (DDBJ) under the accession number PRJDB18745.

### 2.4. Bioinformatics

The raw FASTQ data were processed using the QIIME2 pipeline (version 2023.2). Error correction, quality filtering, chimera removal, and sequence variant calling of the Illumina amplicon sequences were performed using the DADA2 algorithm. The sequence reads were truncated at 250 bp with a quality score of >30. Following the sequence variant calling, singletons and doubletons were removed. A QIIME2-formatted reference database of plant rbcL genes from the NCBI database was developed [27] and used as the benchmark for taxonomy assignment using the q2-feature-classifier plugin for QIIME2.

To evaluate the detection efficiency of the primer pairs for terrestrial plant monitoring, the sequences obtained from each sample were classified into Streptophyta, Chlorophyta, and unassigned categories. Sequences not identified as Streptophyta were excluded from further analysis. The relative frequency of each plant taxon at the family and genus levels within the Streptophyta-classified sequences from each sample was calculated and averaged across the three replicates. The plant taxa at the family and genus levels detected from eDNA were then compared with the list of surrounding terrestrial vegetation based on field surveys. Heatmaps based on relative frequency were generated for each pond, excluding those classified as unassigned at each taxonomic level.

## 3. Results

### 3.1. Identification of Plant Families

**Figure 2:**
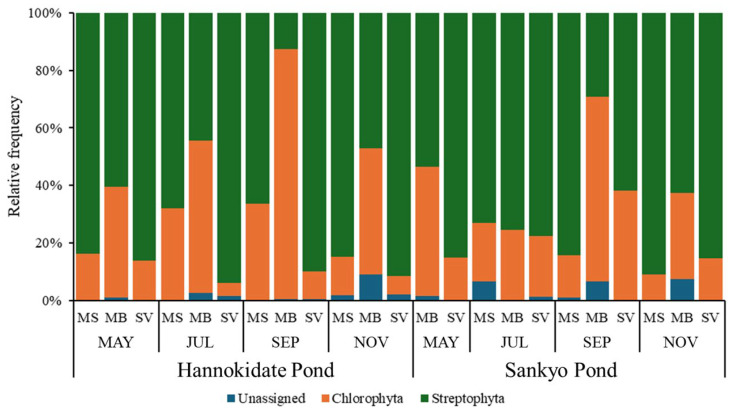
The balance between Chlorophyta and Streptophyta in amplicons obtained from pond water eDNA extracted from the samples sequentially fractionated by 200 µm mesh filters (MS), 5 µm membrane filters (MB), and 0.45 µm Sterivex filters (SV) in that order. H and S stand for Hannokidate Pond and Sankyo Pond, respectively, from which the pond water was collected.

The rbcL primer used in this study amplified 28% of green algal DNA (Chlorophyta) and 70% of terrestrial plant DNA (Streptophyta) as shown in Figure 2. Through the eDNA analysis, a comprehensive taxonomic assessment identified a total of 46 families and 57 genera of plants.

The families with the highest relative frequency were Juglandaceae, followed by Actinidiaceae, Sapindaceae, Rosaceae, Moraceae, Hydrangeaceae, Urticaceae, and Malvaceae. In terms of genus richness, the Pinaceae family exhibited the highest richness with five distinct genera, followed by Solanaceae, Rosaceae, Urticaceae, Fabaceae, Hydrangeaceae, Polygonaceae, and Rutaceae.

The list of plant families and genera in the surrounding vegetation of both ponds, based on field surveys and historical records, along with those detected by eDNA analysis, is shown in Table 1 and Table S1. Upon comparison of the results for each pond individually, the eDNA analysis identified 45 families and 51 genera in Hannokidate Pond, whereas 52 families and 62 genera were recorded by the field survey. Similarly, in Sankyo Pond, eDNA analysis identified 31 families and 30 genera out of the 46 families and 55 genera recorded by the field survey. The shared taxa detected in the eDNA from both ponds were 29 families and 21 genera.

### 3.2. Temporal Shift

**Figure 3:**
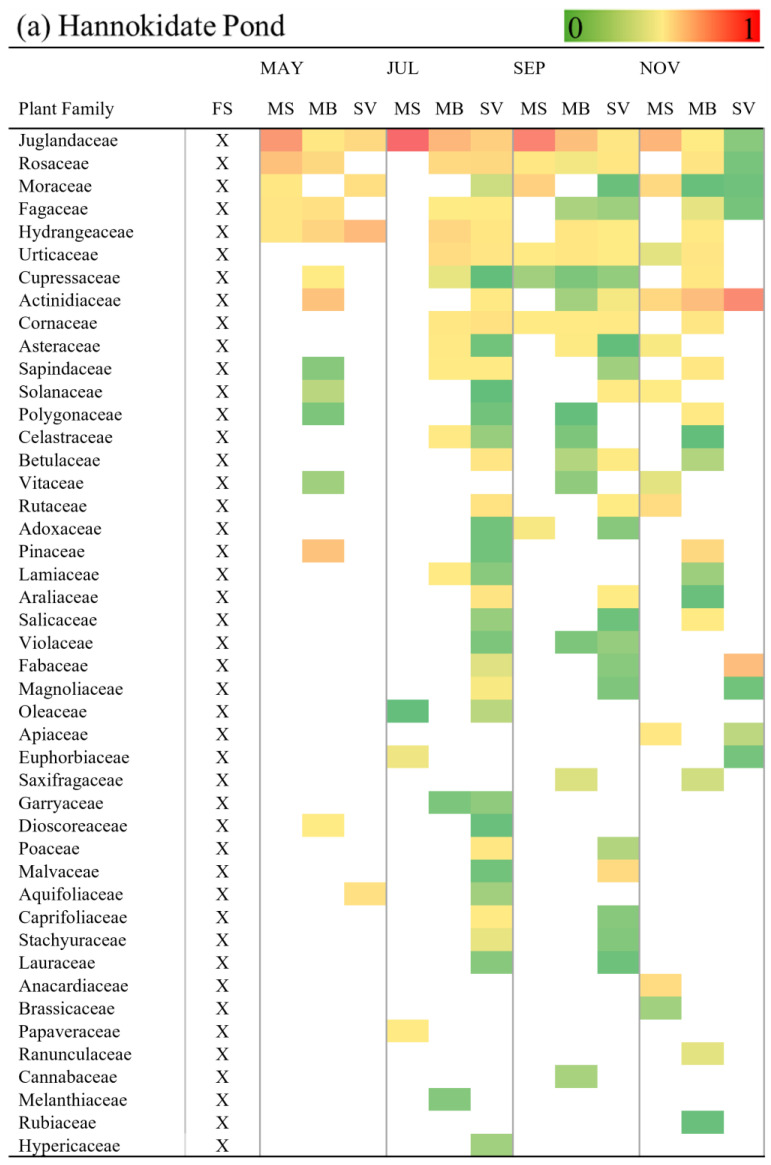

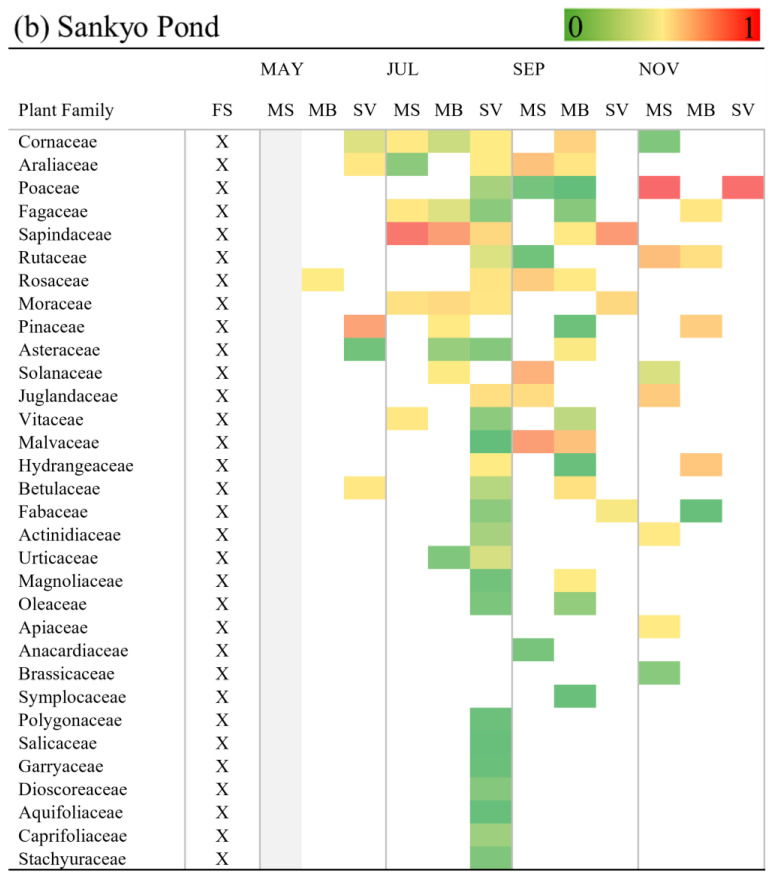
(**a**) Plant families detected in amplicons obtained from Hannokidate Pond water eDNA extracted from the samples sequentially fractionated by 200 µm mesh filters (MS), 5 µm membrane filters (MB), and 0.45 µm Sterivex filters (SV) in that order. (**b**) Plant families detected in amplicons obtained from Sankyo Pond water eDNA extracted from the samples sequentially fractionated by 200 µm mesh filters (MS), 5 µm membrane filters (MB), and 0.45 µm Sterivex filters (SV) in that order. The color represents the relative frequency of each plant family. FS stands for a field survey conducted in November 2023. The observed families are indicated by “X”. Gray color means that no data were available since sufficient amplicons were not obtained.

When comparing the temporal distribution patterns of the plant families and genera identified by the eDNA analysis, the July samples showed the highest number of plant family and genus in both ponds, as 31 genera of 37 families were detected from the Hannokidate Pond water and 24 genera in 28 families were from the Sankyo Pond water (Figure 3 and Figure S1). Subsequently, the September samples exhibited the second highest number of detected plant families and genera, followed by a decrease in detected plant groups in November and May, respectively. This pattern remained consistent across both ponds.

Regarding the specific families detected in Hannokidate Pond, none was exclusively observed in May, while six families were solely observed in July, one in September, and five in November. Similarly, in Sankyo Pond, no families were exclusively detected in May, whereas seven were observed only in July, two in September, and three in November. In both ponds, the Pinaceae family was consistently detected in all four months, the Rutaceae family in July, September, and November, and the Malvaceae family only in July and September. Additionally, the Garryaceae family was detected in July in both ponds, while the Brassicaceae and Apiaceae families were exclusively detected in November (Figure 3). At the genus level, two genera were exclusively detected in the Hannokidate Pond water in May, six in July, three in September, and eight in November. Conversely, in the Sankyo Pond water, none was detected in May, 11 in July, two in September, and three in November (Figure S1).

### 3.3. Detection of Plant Taxa Across Size Fractions

At the family level in Hannokidate Pond, a total of 20 families were detected in the eDNA obtained from the 200 μm mesh filters, 28 from the 5 μm membrane filters, and 36 from the Sterivex filters during the sampling period. Among these, three families (Anacardiaceae, Brassicaceae, and Papaveraceae) were exclusively identified with the 200 μm mesh filters, four (Ranunculaceae, Cannabaceae, Melanthiaceae, and Rubiaceae) with the 5 μm membrane filters, and one (Hypericaceae) with the Sterivex filters (Figure 3a). In Sankyo Pond, a total of 16 families were detected in the eDNA obtained from the 200 μm mesh filters, 20 from the 5 μm membrane filters, and 27 from the Sterivex filters. Among these, three families (Apiaceae, Anacardiaceae, and Brassicaceae) were exclusively identified with the 200 μm mesh filters, one (Symplocaceae) with the 5 μm membrane filters, and seven (Polygonaceae, Salicaceae, Garryaceae, Dioscoreaceae, Aquifoliaceae, Caprifoliaceae, and Stachyuaraceae) with the Sterivex filters (Figure 3b). In Hannokidate Pond, 11 families (Juglandaceae, Rosaceae, Moraceae, Fagaceae, Hydrangeaceae, Urticaceae, Cupressaceae, Actinidaceae, Cornaceae, Asteraceae, and Solanaceae) were detected from the eDNA extracted using any of the filter types, while in Sankyo Pond, eight families (Cornaceae, Araliaceae, Poaceae, Fagaceae, Sapindaceae, Rutaceae, Rosaceae, and Moraceae) were detected from the eDNA extracted using all the filter types. In both ponds, the Brassicaceae and Anacardiaceae families were only detected in the eDNA captured by the mesh filters.

At the genus level, a total of 15 genera were detected in the eDNA obtained from the 200 μm mesh filters, 32 from the 5 μm membrane filters, and 29 from the Sterivex filters in Hannokidate Pond during the sampling period (Figure S1a). In Sankyo Pond, a total of 13 genera were detected in the eDNA obtained from the 200 μm mesh filters, 17 from the 5 μm membrane filters, and 22 from the Sterivex filters (Figure S1b). Common features observed in both ponds include the exclusive identification of the genus *Brassica* using the 200 μm mesh filters and *Cedrus* using the 5 μm membrane filters. Additionally, the genera *Glycine*, *Stachyurus*, *Ilex*, *Phellodendron*, and *Salix* were solely detected with the Sterivex filters. The genera *Morus*, *Purus*, and *Cornus* were consistently detected across all three filter types, whereas *Picea* was identified using both the membrane and Sterivex filters.

When comparing the number of plant families detected at different times, the highest number in Hannokidate Pond was observed in November from eDNA captured by the 200 µm mesh and 5 µm membrane filters, and in July from the Sterivex filters. In Sankyo Pond, the highest number of families was observed in November for the 200 µm mesh, in September for the 5 µm membrane filters, and in July for the Sterivex filters (Figure 3).

## 4. Discussion

### 4.1. Taxonomic Findings

The universal rbcL gene primer sets have shown limited effectiveness in identifying green algae, as noted in previous studies [28]. Instead, their use is generally more suitable for identifying terrestrial plants [29,30]. In this study, an estimated average of 28% of the sequences obtained from the eDNA samples (ranging from a minimum of 4% to a maximum of 87%) were derived from algae (Figure 2), yet sufficient reads identifying terrestrial plants were also obtained. Variations in DNA concentrations from the eDNA samples may be attributed to environmental factors such as temperature, pH, and UV radiation exposure, which are known to impact DNA stability [31], or to the effects of these factors on the growth of green algae [32]. These findings support the utility of the rbcL gene primer set for ecological surveys targeting terrestrial plant richness using eDNA.

In this study, 79% and 63% of the families and genera identified in the field visual survey were detected by the eDNA analysis of the pond water. Given the successful amplification of plant DNA from animal and bird feces in many studies [33,34,35], it is challenging to assess whether these plant DNA origins are relatively recent. However, it can be asserted that all the detected families in the eDNA samples collected in this study, as shown in Table 1, were confirmed to exist in the surrounding area, thereby reflecting the local vegetation. Some taxa were identified only at the family level or were detected but are considered absent in the vicinity, possibly due to misidentification stemming from the short length of sequences. Considering the limitation of amplicon sequencing on the MiSeq platform, it is likely that identification is limited to the family or genus level. However, by using long-read sequencers, it may be possible to design more appropriate primers that exclude algae and enable the identification of plant communities at the species level. Future studies employing long-read sequencing technologies could overcome the current taxonomic limitations, enabling more precise species-level identification, particularly for complex plant communities.

### 4.2. Temporal Distribution Properties

Examining the results temporally, the highest number of identified plants in both ponds was observed in July. Although some plants were identified only in certain months in both ponds, the number of plants identified at both the family and genus levels in July was generally higher than in other months. This seasonal peak is likely influenced by the unique climatic and ecological conditions during this period. In general, vegetation that emerges in May undergoes decomposition as flowers mature, petals fall, and pollen disperses. The flowers, pollen, and fresh branches that are shed or carried by the rainy season and the increase in temperature could have been captured with eDNA. Japan’s climatic conditions include a rainy season between June and July [36]. Rainfall during this season may tear off parts of existing plants, causing them to fall and disperse. Ferreira et al. reported that leaves (72%), fresh twigs (14%), reproductive parts (11%), and other plant parts (3%) fall onto the forest surface, with the highest accumulation occurring in late spring and mid-summer in southeastern Brazil [37]. These findings align with the increased DNA input observed in the ponds during July, suggesting a direct link between seasonal vegetation dynamics and eDNA deposition. They attributed this to the decrease in plant reproductive organs due to high rainfall, increased temperatures, and solar radiation. Another study reported the highest accumulation of plant material in the forest between July and September in Japan [38]. Additionally, many fruits in Japanese forest that are consumed by birds and animals ripen in July [39]. The consumption and subsequent dispersal of these fruits by animals likely contribute to the input of plant DNA to the ponds.

Another important factor is pollen distribution and accumulation. This may explain why the number of detected plant taxa was higher in July. It was reported that pollen amounts increased with rising temperatures, peaking in July in Japan [40]. Similarly, the seasonal dynamics of pollen from some Poaceae family species were examined and it was observed that some species peaked in June–July [41]. In their year-long study, it was reported that many species’ pollen rates peaked in these months [42]. Considering that pollen grains can persist in aquatic environments [43], these studies suggest that seasonal pollen dispersal may enrich the eDNA pool in pond water. In light of this information, it is possible that the seasonal fluctuations in plant eDNA composition observed in the pond water in this study are influenced by events in the terrestrial plant ecosystem, such as plant fragmentation during the rainy season, fruit consumption, and the formation and distribution of pollen.

### 4.3. Evaluation of Sequential Filtration

In studies assessing plant diversity using eDNA, 0.22 µm or 0.45 µm Sterivex filters were commonly employed to capture eDNA, yet there is limited discussion regarding the specific nature of the captured material. In some studies, aimed at testing the functionality of eDNA, additional filters are used to remove non-eDNA particles and larger contaminants, in addition to filters that capture only DNA. For example, a study reported that by separating material according to particle size using sequential filters, larger particles did not decay, while smaller particles were renewed over time [44]. Previous research in the marine ecosystem suggest that the particle size retained by different filter can significantly influence the detection of certain taxa, as smaller particles might contain degraded DNA fragments, while larger particles may retain intact tissues [45,46]. However, there has been little discussion to date regarding the forms, origins, and transport pathways of eDNA derived from terrestrial plant ecosystems.

The sequential filtration of the plant-derived materials revealed that plant eDNA in pond water exhibits distinct characteristics across different sizes. Most of the plants identified in the eDNA samples collected using the 200 µm mesh filters were also identified in those collected using the other filters. The plants identified solely in the eDNA samples collected using the 200 µm mesh filters (Brassicaceae and Anacardiaceae) were insect-pollinated and possess seeds enclosed within capsules or pods [47,48,49]. The identification of these plant families exclusively in the samples captured by the 200 µm mesh filters suggests that seeds or fragmented plant parts produced during September and November may have been transported to the pond water through rain-induced water or wind dispersal [50,51]. The two other filters capturing smaller fractions yielded more plant taxa eDNA, but the 5 µm membrane filters tended to capture eDNA containing a broader range of plant debris compared to the Sterivex filters in May and November. Considering that pollen grains typically range in size from 7 to 100 µm [52], and that the increase in detected plant taxa aligns with the pollen dispersal periods in early summer and autumn [53], it is suggested that the eDNA captured by the 5 µm membrane filters might be influenced by pollen particles. In contrast, numerous plant DNAs were detected in the eDNA samples collected using the Sterivex filters in July and September. This pattern may be attributed to the enhanced activity of organic matter decomposers during the warmer summer months [54,55], leading to the finer fragmentation of plant debris and increased detection of smaller DNA particles. Currently, it is inconclusive whether the differences in plant taxa detected across the eDNA fractions obtained by the sequential filtration reflect forest phenology, such as pollen dispersal, or the organic matter decomposition processes.

Although there have been reports on the movement of eDNA and nucleic acids within ponds [32], the pathways from terrestrial sources to this process remain largely unclear. Previous studies have demonstrated that genetic material transported into aquatic systems via abiotic and biotic vectors, such as rainfall, wind, and animal activity, can reliably represent the surrounding terrestrial ecosystem [30,31,36]. Moreover, the importance of understanding the ecology of eDNA, including the origin, state, transport, and fate of extraorganismal genetic material [30], as well as plant–animal interactions [56], has been emphasized. In this study, it was also demonstrated that the eDNA in pond water, composed of various forms of plant-derived materials, reflects the surrounding vegetation. However, a deeper understanding of these characteristics could facilitate the monitoring of broader dynamics within terrestrial plant ecosystems, extending beyond species composition. Therefore, future research should aim to clarify the origin and pathways of plant eDNA, quantify these pathways, and investigate the appropriate fractionation of pollen and plant remains. This approach could enable low-cost, long-term monitoring of critical ecological events, such as mast flowering, mast seeding, and mass dieback, in addition to phenological events, through pond water eDNA analysis.

## 5. Conclusions

This study demonstrates that the plant taxa detected in pond water eDNA were generally consistent with the surrounding terrestrial plant ecosystem, supporting our hypothesis that pond water eDNA can reflect the surrounding terrestrial plant communities. This finding suggests that eDNA could serve as a valuable tool for monitoring plant diversity in forest ecosystems. Furthermore, it was shown that plant eDNA in pond water exhibited distinct characteristics based on particle size. Future research should focus on enhancing the resolution of plant taxonomy identification and elucidating the origins and pathways of plant-derived eDNA. Such advancements could enable the monitoring of various ecological events in terrestrial plant ecosystems through eDNA, contributing to more comprehensive and informative forest ecosystem monitoring.

## Figures and Tables

**Table 1:** Plant families likely present in the surroundings based on field surveys (visual inspection in 2023 and historical records, denoted as FS) and those detected through eDNA analysis (denoted as DNA) during the sampling periods. Presence is indicated by “X”.

			Hannokidate		Sankyo	
Group	Order	Family	FS	DNA	FS	DNA
GYMNOSPERMS	Cupressales	Cupressaceae	X			
	Pinales	Pinaceae	X	X	X	X
ANGIOSPERMS						
Basal angiosperms	Laureales	Lauraceae	X	X	X	
	Piperales	Saururaceae	X			
	Magnoliales	Magnoliaceae	X	X		
Monocots	Alismatales	Araceae			X	
	Asparagales	Asparagaceae	X			
		Orchidaceae			X	
	Discoreales	Dioscoreaceae	X	X	X	X
	Liliales	Colchicaceae			X	
		Melanthiaceae	X	X		
		Smilacacear	X		X	
	Poales	Cyperaceae			X	
		Poaceae		X		X
Eudicots	Ranunculales	Berberidaceae			X	
		Lardizabalaceae	X		X	
		Papaveraceae	X	X		
		Ranunculaceae	X	X	X	
Core eudicots						
Superrosids	Saxifragales	Saxifragaceae	X	X	X	
Rosids	Vitales	Vitaceae	X	X	X	X
	Fabales	Fabaceae		X		X
	Fagales	Betulaceae	X		X	
		Fagaceae	X	X	X	X
		Juglandaceae	X			
	Rosales	Cannabaceae	X	X		
		Hydrangeaceae	X	X	X	X
		Moraceae	X	X	X	X
		Rosaceae	X	X	X	X
		Urticaceae	X	X	X	X

## Data Availability

Raw sequence reads are deposited in the DNA Data Bank of Japan (DDBJ) under the accession number PRJDB18745. Sample metadata are also available in the DDBJ under the same accession number PRJDB18745.

## Supplementary Materials

The following supporting information can be downloaded at: https://www.mdpi.com/article/10.3390/biology14010062/s1, Figure S1: (a) Plant genera detected in amplicons obtained from pond water eDNA samples collected from Hannokidate Pond. Samples were sequentially fractionated using 200 μm mesh filters (MS), 5 μm membrane filters (MB), and 0.45 μm Sterivex filters (SV). The color represents the relative frequency of each plant genus. (b) Plant genera detected in amplicons from Sankyo Pond, analyzed under the same fractionation process. The field survey (FS) conducted in November 2023 marks observed genera with “X”, while unobserved genera are left blank. Gray color indicates unavailable data due to insufficient amplicons; Table S1: Plant genera likely present in the surroundings based on field surveys (visual inspection in 2023 and historical records [57,58,59], denoted as FS) and those detected through eDNA analysis (denoted as DNA) during the sampling periods. Presence is indicated by “X”.
