# Supplementary material for: Pond Water eDNA Reflects Broad Consistency with Surrounding Terrestrial Plant Ecosystems
Source: Biology (Basel). 2025 Jan 13;14(1):62. doi: 10.3390/biology14010062 (PMC11762844; doi:10.3390/biology14010062)
Supplement: Supplementary file 1 [file biology-14-00062-s001.zip › biology-3396145-supplementary.pdf]

Pond Water eDNA Reflects Broad Consistency with Surrounding Terrestrial Plant Ecosystems

Duygu BOZDOGAN [1], Shogo TAKIZAWA [2], Norihiro FURUKORI [3], Kosuke HOMMA [3], Harue ABE [3], Hitoshi SAKIO [3], Naoki HARADA [4], Kazuki SUZUKI [4,*]

1 Graduate School of Science and Technology, Niigata University, 950-2181 Niigata, Japan;

2 Faculty of Agriculture, Niigata University, 950-2181 Niigata, Japan;

3 Sado Island Center for Ecological Sustainability, Niigata University, 952-0103 Niigata, Japan;

4 Institute of Science and Technology, Niigata University, 950-2181 Niigata, Japan;

* Correspondence: suzukik@agr.niigata-u.ac.jp

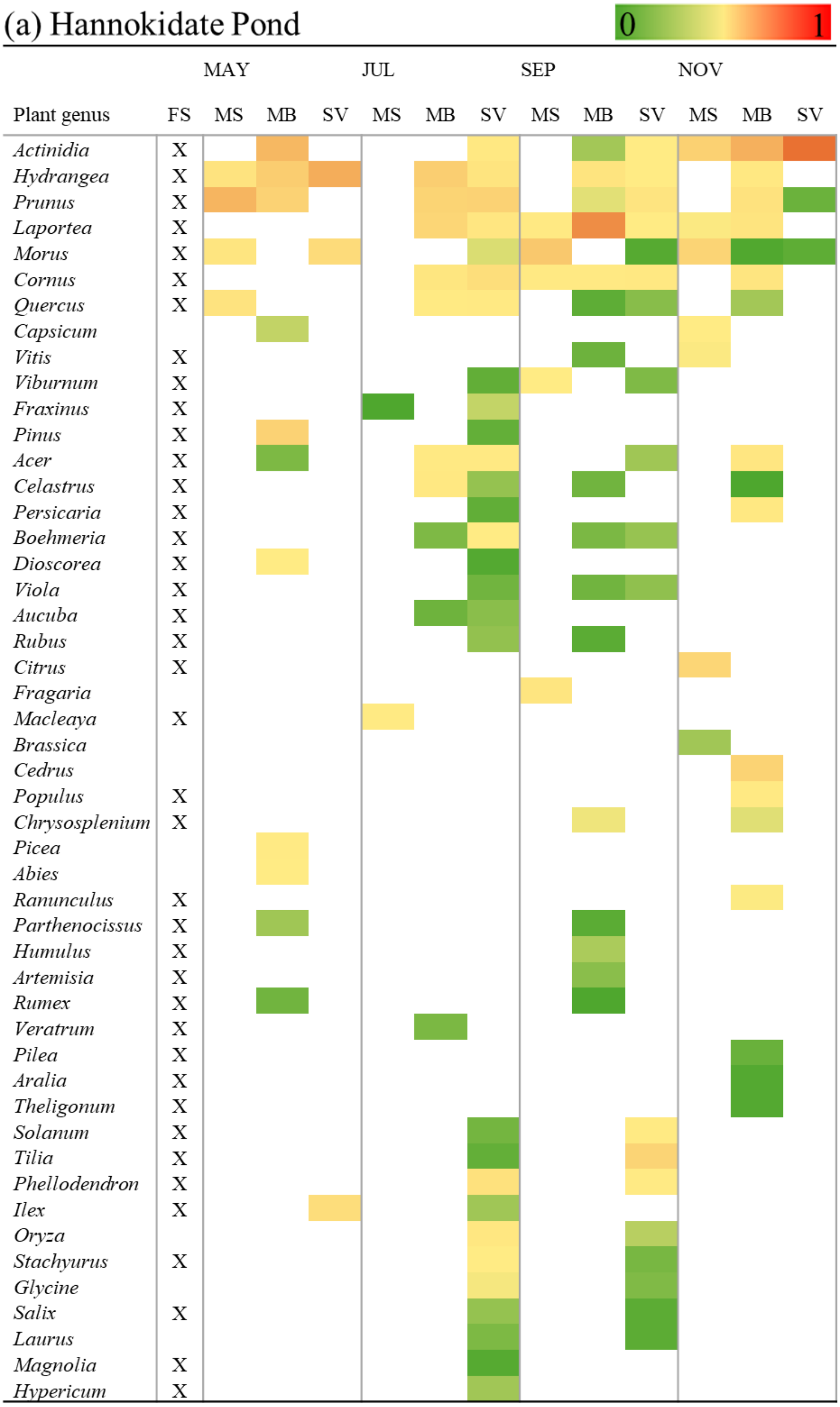
(a) Hannokidate Pond
0
1
MAY
JUL
SEP
NOV
Plant genus
FS
MS
MB
SV
MS
MB
SV
MS
MB
SV
MS
MB
SV
Actinidia
Hydrangea
Prunus
Laportea
Morus
Cornus
Quercus
Capsicum
Vitis
Viburnum
Fraxinus
Pinus
Acer
Celastrus
Persicaria
Boehmeria
Dioscorea
Viola
Aucuba
Rubus
Citrus
Fragaria
Macleaya
Brassica
Cedrus
Populus
Chrysosplenium
Picea
Abies
Ranunculus
Parthenocissus
Humulus
Artemisia
Rumex
Veratrum
Pilea
Aralia
Theligonum
Solanum
Tilia
Phellodendron
Ilex
Oryza
Stachyurus
Glycine
Salix
Laurus
Magnolia
Hypericum

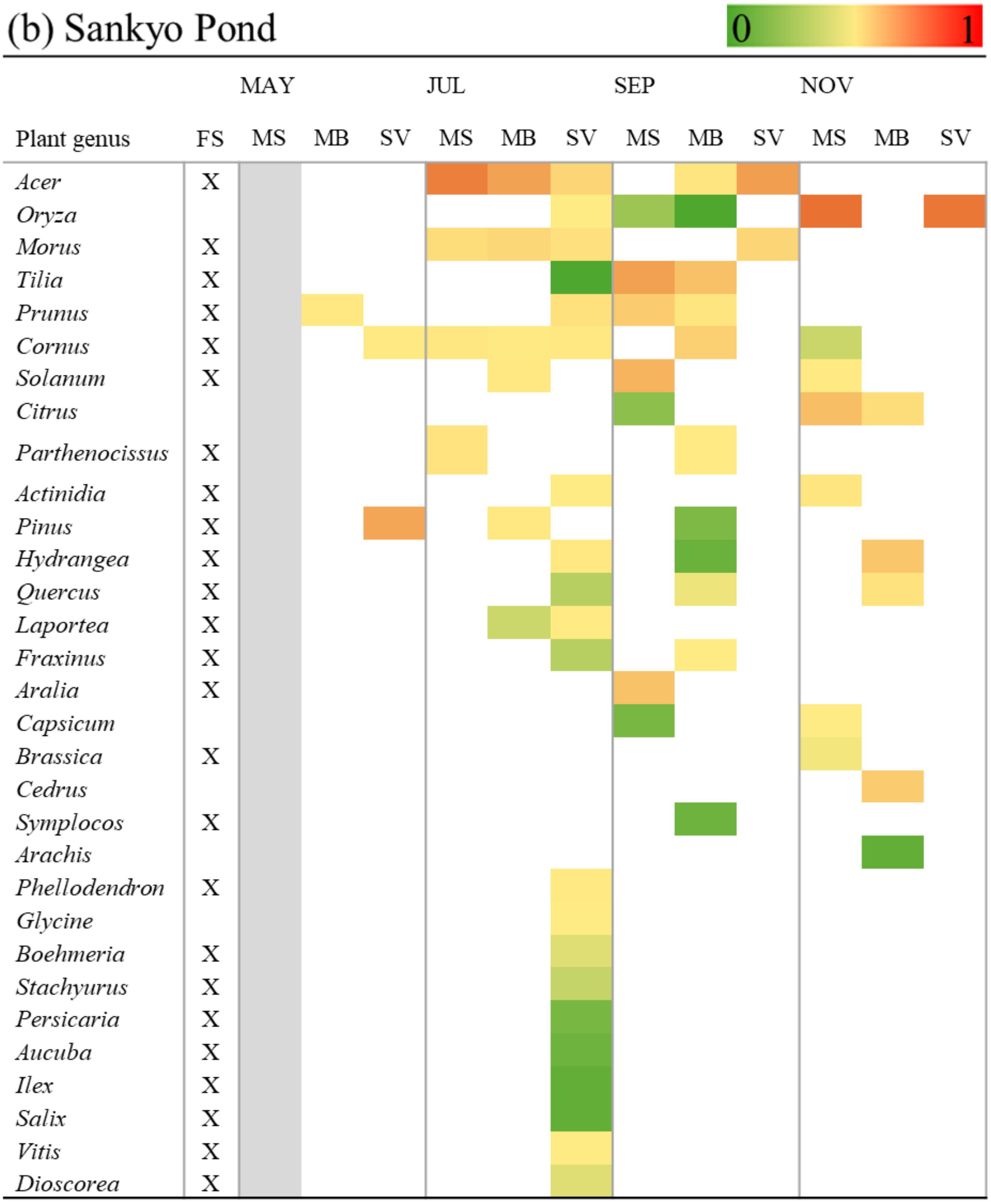

Fig S1. Plant genera detected in amplicons obtained from pond water eDNA extracted from the samples sequentially fractionated by 200 µm mesh filters (MS), 5 µm membrane filters (MB), and 0.45 µm Sterivex filters (SV) in this order. The color represents the relative frequency of each plant genera. FS stands for a field survey conducted in November 2023. The observed genera are indicated by “X” and those not observed are left blank. Gray color means no data were available since sufficient amplicons were not obtained.

Table S1. Plant genera likely present in the surroundings based on field surveys (visual inspection in 2023 and historical records [57-59]; denoted as FS) and those detected through eDNA analysis (denoted as DNA) during the sampling periods. Presence is indicated by “X”.

			Hannokidate		Sankyo		
Order	Family	Genus	FS	DNA	FS	DNA	Species distributed in the area
**GYMNOSPERMS**							
Cupressales	Cupressaceae	*Cryptomeria*	X				*C. japonica*
		Thujopsis	X				*T. dolabrata var. hondae*
Pinales	Pinaceae	*Pinus*	X	X	X	X	*P. densiflora, P. parviflora var. pentaphylla*
		Abies		X			*A.firma* (distributed in the central / southern part of the island)
		Cedrus		X		X	- (not distributed, plantation only)
		Picea		X			- (not distributed, plantation only)
**ANGIOSPERMS**							
**Basal angiosperms**							
Laureales	Lauraceae	*Laurus*		X			- (not distributed, plantation only)
		Lindera	X		X		*L. umbellata var. membranacea*
Piperales	Saururaceae	*Houttuynia*	X				*H. cordata*
Magnoliales	Magnoliaceae	*Magnolia*	X	X			*M. obovata, M. salicifolia*
**Monocots**							
Alismatales	Araceae	*Arisaema*			X		*A. japonicum, A. ovale var. sadoense*
Asparagales	Asparagaceae	*Polygonatum*	X				*P. falcatum, P. odoratum*
	Orchidaceae	*Calanthe*			X		*C. discolor*

Table S1 (cont.)

			Hannokidate		Sankyo		
Order	Family	Genus	FS	DNA	FS	Order	Species distributed in the area
Discoreales	Dioscoreaceae	*Dioscorea*	X	X	X	X	*D. japonica, D. tokoro* etc.
Liliales	*Colchicaceae*	*Disporum*			X		*D. sessile*、*D. smilacinum*
	Melanthiaceae	*Veratrum*	X	X			*V. maackii*
	Smilacacear	*Smilax*	X		X		*S. china, S. riparia*
Poales	Cyperaceae	*Carex*			X		*C. multifolia* etc.
	Poaceae	*Oryza*		X		X	- (not distributed, plantation only)
**Eudicots**							
Ranunculales	Berberidaceae	*Caulophyllum*			X		*C. robstum*
	Lardizabalaceae	*Akebia*	X		X		*A.quinata*
	Papaveraceae	*Macleaya*	X	X			*M. cordata*
	Ranunculaceae	*Aconitum*	X	X			*A. japonicum*
		Cimicifuga	X				*C. simplex, C. biternata*
		Clematis			X		*C. apiifolia, C. terniflora* etc.
		Ranunculus	X	X			*R. silerifolius, R. japonicus*
**Core eudicots**							
**Superrosids**							
Saxifragales	Saxifragaceae	*Chrysosplenium*	X	X			*C.　fauriei*
		Tiarella			X		*T. polyphylla*

Table S1 (cont.)

Order	Family	Genus	Hannokidate FS	Hannokidate DNA	Sankyo FS	Sankyo Order	Species distributed in the area
**Rosids**							
Vitales	Vitaceae	*Vitis*	X	X	X	X	*V. coignetiae, V. flexuosa*
		Parthenocissus	X	X	X	X	*P. tricuspidata*
Fabales	Fabaceae	*Glycine*		X		X	- (not distributed, plantation only)
Fagales	Betulaceae	*Alnus*	X				*A. hirsuta*
		Corylus			X		*C. sieboldiana*
	Fagaceae	*Castanea*			X		*C. crenata*
		Quercus	X	X	X	X	*Q. crispula, Q. dentata, Q. serrrata*
	Juglandaceae	*Juglans*	X				*J. mandshurica var. sieboldiana*
		Pterocarya	X				*P. rhoifolia*
Rosales	Cannabaceae	*Humulus*	X	X			*H. japonicus*
	Hydrangeaceae	*Hydrangea*	X	X	X	X	*H. serrata var. yesoensis, H. petioralis, H. panivulata*
	Moraceae	*Morus*	X	X	X	X	*M. australis*
	Rosaceae	*Cerasus* (*Prunus*)			X		*C. sargentii* var. *sargentii, C. leveilleana*
		Padus (*Prunus*)	X		X		*P. grayana*
		Filipendula	X				*F. camtschatica*
		Fragaria		X			- (not distributed, plantation only)
		Prunus	X	X	X	X	(subgenus *Padus*) *Prunus grayana, Prunus ssiori*
		Rubus	X	X	X		*R. crataegifolius*、*R. palmatus, R. pectinellus* etc.
	Urticaceae	*Pilea*	X	X			*P. pumila*
		Boehmeria	X	X	X	X	*B. silvestrii, B. platanifolia*

Table S1 (cont.)

			Hannokidate		Sankyo		
Order	Family	Genus	FS	DNA	FS	Order	Species distributed in the area
		Elatostema			X		*E. involucratum* etc.
		Laportea	X	X	X	X	*L. cuspidata, L. bulbifera*
**Rosids or Malvidae**							
Celastrales	Celastraceae	*Celastrus*	X	X			*C. orbiculatus, C. orbiculatus var. strigillosus*
Malpighiales	Euphorbiaceae	*Mallotus*	X				*M. japonicus*
		Neoshirakia			X		*N. japonica*
	Hypericaceae	Hypericum	X	X			*H. erectum*
	Salicaceae	*Populus*	X	X			*P. tremula var. sieboldii*
		Salix	X	X	X	X	*S. caprea, S. udensis* etc.
	Violaceae	*Viola*	X	X			*V. grypoceras var. grypoceras, V. vaginata* etc.
**Malvidae**							
Brassicales	Brassicaceae	*Brassica*		X	X	X	*B. napus* etc. (plantation)
Crossosomatales	Stachyuraceae	*Stachyurus*	X	X	X	X	*S. praecox*
Malvales	Malvaceae	*Tilia*	X	X	X	X	*T. japonica*
Sapindales	Anacardiaceae	*Rhus*	X		X		*R .javanica*
	Rutaceae	*Citrus*		X		X	- (not distributed, plantation in the southern part of the island)
		Phellodendron	X	X	X	X	*P. amurense*
	Sapindaceae	*Acer*	X	X	X	X	*A.palmatum, A. mono, A. rufinerve*
	Staphyleaceae	*Staphylea*	X		X		*S. bumalda*

Table S1 (cont.)

Order	Family	Genus	Hannokidate FS	Hannokidate DNA	Sankyo FS	Sankyo Order	Species distributed in the area
**Superasterids**							
Caryophyllales	Polygonaceae	*Persicaria*	X	X	X	X	*P. nodosa, P. nodosa*
		Rumex	X	X			*R. jaonicus*
**Asterids**							
Cornales	Cornaceae	*Cornus*	X	X	X	X	*C. controversa* var. *controversa*, *C. macrophylla*
Ericales	Pentaphylacaceae	*Eurya*			X		*E. japonica*
	Pentaphylacaceae	*Eurya*			X		*E. japonica*
	Styracaceae	*Styrax*			X		*S. japonicus*
	Symplocaceae	*Symplocos*			X	X	*S. sawafutagi*
Garryales	Garryaceae	*Aucuba*	X	X	X	X	*A. japonica var. borealis*
Gentianales	Rubiaceae	*Theligonum*	X	X			*T. japonicum*
Solanales	Solanaceae	*Capsicum*		X		X	- (not distributed, plantation only)
		Solanum	X	X	X	X	*S. lyratum*
Lamiales	Lamiaceae	*Callicarpa*			X		*C. japonica*
		Clerodendrum	X				*C. trichotomum*
		Clinopodium	X				*C. gracile, C. micranthum* etc.
	Oleaceae	*Fraxinus*	X	X	X	X	*F. lanuginosa, F. longicuspis* etc.
		Ligustrum	X		X		*L. tschonoskii* etc.
Apiales	Araliaceae	*Aralia*	X	X	X	X	*A. elata, A. cordata*
		Hedera			X		*H. rhombea*
		Kalopanax			X		*K. septemlobus*
	Apiaceae	*Angelica*	X	X	X	X	*A. pubescens*

Table S1 (cont.)

Order	Family	Genus	Hannokidate FS	Hannokidate DNA	Sankyo FS	Sankyo Order	Species distributed in the area
Aquifoliales	Aquifoliaceae	*Ilex*	X	X	X	X	*I. leucoclada, I. macropoda, I. crenata var. radicans* etc.
	Helwingiaceae	*Helwingia*	X		X		*H. japonica*
Asterales	Asteraceae	*Cirsium*			X		*C. nipponicum ver. sadoense*
		Petasites	X				*P. japonicus*
		Artemisia	X				*A. indica var. maximowiczii*
		Parasenecio	X		X		*P. adenostyloides, P. delphiniifolia* etc.
Ericales	Actinidiaceae	*Actinidia*	X	X	X	X	*A. polygama*
Dipsacales	Caprifoliaceae	*Weigela*			X		*W. hortensis*
	Viburnaceae	*Viburnum*	X	X	X		*V. furcatum, V. dilatatum, V. sieboldii Miq. var. obovatifolium*
